# β-Lactam concentrations monitored in the early phase of community-acquired sepsis in the intensive care unit

**DOI:** 10.1093/jac/dkaf401

**Published:** 2025-10-28

**Authors:** Karolina Liljedahl Prytz, Emma Kryss, Joakim Oxelbark, Jan Källman, Kristofer F Nilsson, Martin Sundqvist, Johanna Savilampi

**Affiliations:** Department of Infectious Diseases, Faculty of Medicine and Health, Örebro University, Örebro, Sweden; School of Medical Sciences, Örebro University, Örebro, Sweden; School of Medical Sciences, Örebro University, Örebro, Sweden; Department of Anesthesiology and Intensive Care, Capio St. Göran Hospital, Stockholm, Sweden; Faculty of Medicine and Health, Örebro University, Örebro, Sweden; Department of Laboratory Medicine, Faculty of Medicine and Health, Örebro University, Örebro, Sweden; Department of Infectious Diseases, Faculty of Medicine and Health, Örebro University, Örebro, Sweden; School of Medical Sciences, Örebro University, Örebro, Sweden; Department of Cardiothoracic and Vascular Surgery, Faculty of Medicine and Health, Örebro University, Örebro, Sweden; Department of Laboratory Medicine, Clinical Microbiology, Faculty of Medicine and Health, Örebro University, Örebro, Sweden; Department of Anesthesiology and Intensive Care, Faculty of Medicine and Health, Örebro University, Örebro, Sweden

## Abstract

**Objectives:**

Optimal antibiotic treatment is important in the treatment of sepsis. However, patients with sepsis are at risk of suboptimal antibiotic concentrations. This study aimed to evaluate β-lactam antibiotic concentrations during the first 48 h in patients with community-acquired sepsis admitted to the ICU, and to identify variables associated with antibiotic concentrations that were too low or too high.

**Methods:**

This prospective, observational, single-centre study included patients aged ≥18 years with a high likelihood of infection, a SOFA score of ≥2p, planned β-lactam antibiotic treatment, and ICU admission. The exclusion criteria were ongoing antibiotic treatment and/or nosocomial infections. β-Lactam concentrations were measured up to seven times during the first 48 h. The estimated trough concentrations were divided by the predetermined MIC to generate MIC-multiples for comparison. Patients were allocated to three groups based on the MIC-multiple (MIC× < 1, 1–8 or >8).

**Results:**

Fifty patients were included, with a median of seven samples per patient (257 samples). The group with MIC-multiples of <1 (*n* = 16) was associated with younger age, lower Charlson comorbidity index, Simplified Acute Physiology Score 3, creatinine concentration, and need for noradrenaline. The group with MIC-multiples of >8 (*n* = 15) had higher creatinine and noradrenaline levels.

**Conclusions:**

ICU patients with sepsis are at risk of either too low or too high antibiotic concentrations, and specific patient characteristics may be predictable. Therapeutic drug monitoring in combination with model-informed precision dosing may also help to optimize antibiotic dosing in the early phase of community-acquired sepsis to prevent treatment failure and toxicity.

## Introduction

Patients with sepsis and/or septic shock exhibit significant variations in age, illness severity, underlying health conditions, sources of infection, and treatment outcomes.^1^ Sepsis, particularly septic shock, can cause severely altered haemodynamics, fluid overload, renal and/or liver dysfunction, and the need for organ support, potentially resulting in suboptimal antibiotic exposure at infection sites.^2^

β-Lactams are the most frequently used class of antibiotics in the ICU. Antibiotics in this class are hydrophilic, and their pharmacokinetics may be altered by pathophysiological changes in sepsis and septic shock, such as reduced pH.^3,4^ Because hydrophilic antibiotics mainly distribute in interstitial fluid, standard dosing may cause subtherapeutic concentrations in sepsis.^5^ Several studies have described difficulties in attaining concentrations to reach the desired time above the MIC in critically ill patients.^3,6–9^ Risk factors for non-attainment during β-lactam therapy include augmented renal clearance,^3,6,10^ intermittent dosing regimens,^6^ male sex and high BMI.^7^ In addition to underdosing, the altered pathophysiology of septic shock increases the risk of overdosing, potentially leading to toxic effects.^11,12^ This pharmacokinetic variability encourages individualizing β-lactam dosing via therapeutic drug monitoring (TDM).^7^

β-Lactam antibiotics show time-dependent activity, where efficacy depends on maintaining free antibiotic concentrations above the MIC (*fT*_>MIC_).^13,14^ A consensus on the optimal MIC target or the minimum time above this MIC to enhance survival has, to our knowledge, not been obtained.

Using the upper limit of the WT distribution of a relevant organism, MIC_ECOFF_, or the MIC breakpoint to define the susceptible (S) or indeterminate (I) (MIC_S/I_) category for a specific antibiotic may underestimate target attainment, and it has been suggested that the MIC based on the causative bacteria (MIC_Actual_) might be a suitable alternative.^10,15^ However, not all patients with sepsis have positive blood cultures, which consequently limits the utility of the MIC_Actual_. Therefore, using MIC_ECOFF_ or MIC_S/I_ for a species relevant to the infection type and the antibiotic used have been argued to be relevant in clinical practice.^16^ From a practical point of view it could be argued that MIC thresholds in line with the clinical breakpoints used in everyday reporting could be relevant as these should reflect clinical outcome. Previous studies suggest that a concentration of 1–4× MIC for 100% of the dosing interval is a reasonable target,^7^ whereas a concentration of >4× MIC may maximize bactericidal activity and reduce antibiotic resistance.^8,13^ National recommendations in France even suggest a concentration of 4–8× MIC for 100% of the dosing interval to optimize bacteriological and clinical responses in intensive care patients.^17^

Earlier studies included patients in the ICU with both nosocomial and community-acquired sepsis, typically measuring β-lactam concentrations after steady state or after the third dose.^5,7,18^ We identified a knowledge gap regarding β-lactam concentrations in community-acquired sepsis during the most acute phase of infection.

The primary aim of this study was to evaluate β-lactam concentrations during the first 48 h in ICU-treated patients with community-acquired sepsis, and to identify variables associated with low (<1× MIC) or high (>8× MIC) antibiotic concentrations using the EUCAST clinical breakpoint for S or I to define MIC values for target attainment.

## Materials and methods

### Ethics

This study was approved by the Regional Ethical Review Board of Uppsala, Sweden (DNR 2018/121) with an amendment (DNR 2024-07125-02). Written informed consent was obtained from patients or relatives at inclusion.

### Study design and setting

This prospective, observational, single-centre study was conducted at the Örebro University Hospital, Sweden, a tertiary care hospital with approximately 65 000 emergency department (ED) visits annually. The ICU is a mix of surgical and medical units. The study ran from February 2019 to July 2024, with a pause between March and October 2020 due to COVID-19.

### Participants

Inclusion criteria were age of ≥18 years, high likelihood of infection, SOFA score of ≥2, planned β-lactam treatment, and need for intensive care directly from ED. Infection was confirmed through symptoms and medical history, blood sampling/biochemical markers, X-ray and clinical manifestations such as erysipelas. Exclusions were ongoing antibiotic treatment, lack of written informed consent, and nosocomial infections. Sepsis was diagnosed per Sepsis-3 criteria.^19^

### Antibiotic treatment

β-Lactam antibiotics were chosen by the ED physician per clinical routine. Doses were administered as cefotaxime 1 g q8 h, piperacillin/tazobactam 4 g/0.5 g q8 h, meropenem 1 g q8 h or benzylpenicillin 3 g q8 h. Doses were adjusted individually based on illness severity, infection site, creatinine level, antibiotic choice and estimated glomerular filtration rate (eGFR).^20^ In instances where patients were required to switch antibiotics during the study period, the antibiotic administered for the greatest duration within the initial 48 h was selected to prevent duplicate analysis of the same sample. Based on the clinical routine in the ICU, an infectious disease consultant was involved in the patient management post-inclusion. All doses were administered as infusions for 30 min.

### Clinical data

Baseline data included age, sex, comorbidities [Charlson comorbidity index (CCI)],^21^ isolated pathogens, SOFA, Simplified Acute Physiology Score 3 (SAPS 3) and antibiotic choice, retrieved from hospital records. SOFA scores were measured at ICU admission and at 24 and 48 h. Laboratory results included WBC count, creatinine and lactate levels. The administration of vasoactive drugs (length and dose), mechanical ventilation, continuous renal replacement therapy (CRRT), final diagnosis of infection, ICU length of stay (LOS) and 30 day mortality were recorded.

### Sample collection

Two sets of blood cultures were collected aseptically on admission and after 48 h if still in ICU. All patients had an arterial or central venous line. Blood samples for β-lactam levels were taken at admission (0) and 3, 6, 12, 24, 36 and 48 h in sodium heparin tubes, immediately transported to the laboratory and centrifuged for 7 min at 2400 × **g**. Plasma was aliquoted into 2 mL cryotubes and frozen at −80°C. The samples were analysed in batches.

### Antibiotic concentration measurements

Total plasma concentrations were analysed using a fully validated method based on liquid chromatography combined with tandem mass spectrometry (LC-MS/MS) with matrix-matched calibrators and a protein precipitation approach (Supplement S1, available as Supplementary data at JAC Online).

The timing of β-lactam antibiotic concentration measurements was aligned with other blood sampling timepoints, independent of the antibiotic administration schedule, to be able to follow the clinical course of the patient. Consequently, sampling did not occur at pharmacokinetically optimal intervals, such as mid-dose or pre-dose (trough) periods. The delta time, defined as the interval between antibiotic dose administration and the subsequent concentration measurement, was recorded in hours, irrespective of the dose sequence or time of day.

### Estimated trough values and calculation of ‘MIC-multiples’

**Figure 1.**
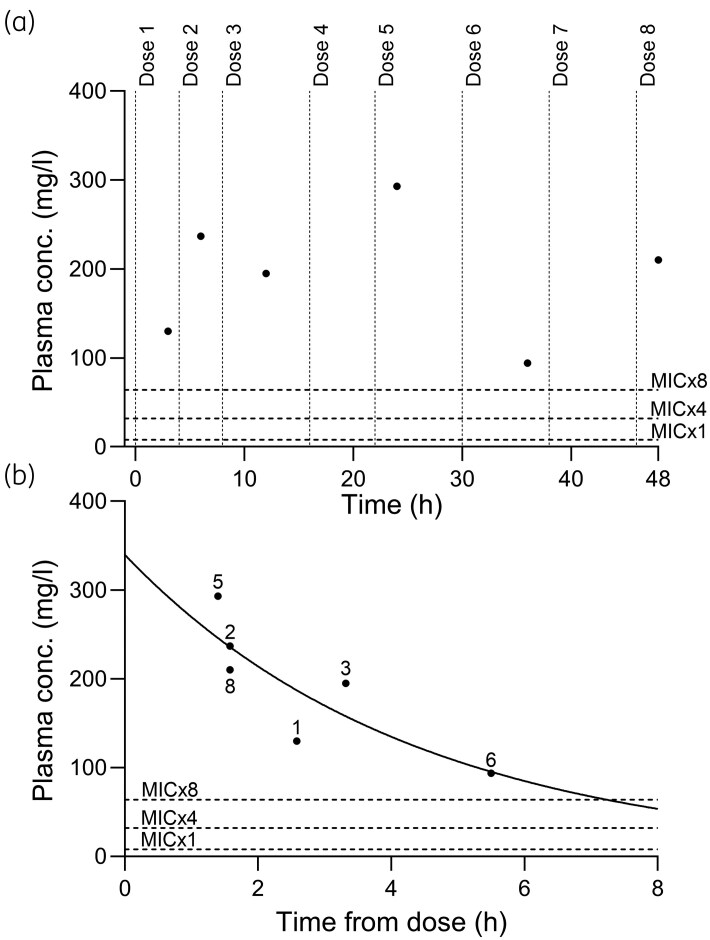
(a) An example of a patient’s sampling schedule during the 48 h of the study. Piperacillin/tazobactam was administered as 4 g/0.5 g q8 h with a bolus dose in the first interval. Antibiotic concentration measurements (six measurements in this case) are displayed as dots. Piperacillin/tazobactam doses are presented as vertical dotted lines, eight doses in total. Target plasma concentration levels are shown in graphs as dotted lines at ×1 MIC, ×4 MIC and ×8 MIC (in this case 8 mg/L for Enterobacterales) according to EUCAST Clinical Breakpoints Table 14.0. (b) The same patient as in (a), now presented with the same measurements of plasma concentrations but shown in relation to delta time (time after the closest previous antibiotic administration). Each measurement is accompanied by the number indicating the dose (chronological) that the delta time is related to. The estimation of trough concentration for the designation of the patient into an MIC multiple group was calculated using *C* = *C*_0_ × *e* (−*k* × *t*). In this case, the estimation rendered the patient in the group with MIC-multiple between 1 and 8.

Since the antibiotic concentration sampling schedule was not at trough timepoints, trough values were estimated based on measured concentrations. The estimated trough concentration was calculated based on each patient’s β-lactam concentration measurements using *C* = *C*_0_ × *e*^(−*k×t*)^, where *C*_0_ is the concentration at time 0, *k* is the rate constant for elimination, and *t* is the delta time (Figure 1). To obtain a reliably estimated value from the calculations, it was necessary to include at least three concentration measurements.

As not all patients had positive blood cultures, we did not use MIC_Actual_; instead, the MICs used for the calculations were based on the MIC_S/I_ (i.e. the highest MIC considered susceptible to increased exposure according to EUCAST Clinical Breakpoint Table version 14.0) based on the following combinations: piperacillin/tazobactam and Enterobacterales, 8 mg/L; cefotaxime and Enterobacterales, 2 mg/L; benzylpenicillin and *Streptococcus pneumoniae*, 2 mg/L; and meropenem and *Pseudomonas aeruginosa*, 8 mg/L. As an addition, and for comparison, calculations were also carried out on MIC_ECOFF_. These were based on the following combinations: piperacillin/tazobactam and Enterobacterales, 8 mg/L; cefotaxime and Enterobacterales, 0.25 mg/L; benzylpenicillin and *Streptococcus pneumoniae*, 0.06 mg/L; and meropenem and *Pseudomonas aeruginosa*, 2 mg/L.^22^

To compare the different antibiotics, the estimated trough concentration was divided by the MIC_S/I_, as defined above, resulting in an MIC-multiple at the trough for each patient. The patients were then divided into three groups based on the MIC-multiple (<1× MIC, 1–8× MIC or >8× MIC) where ‘<1× MIC’ indicated patients were estimated to not reach concentrations above the MIC for the full dosing interval, while ‘>8×MIC’ indicated patients with concentrations of >8 times the MIC throughout the dosing interval.

### Statistical analysis

Categorical data are reported as counts and percentages and were evaluated by the chi-squared test, or Fisher’s exact test when appropriate. Normality of the distribution was tested by the Shapiro–Wilk test. Non-normally distributed continuous variables were presented as medians and IQRs and evaluated by the Kruskal–Wallis test followed by a *post hoc* multiple comparison by the Mann–Whitney *U*-test (including a Bonferroni correction). Normally distributed continuous variables are presented as means and standard deviations (±SD) and were analysed by one-way ANOVA and Tukey’s honest significant difference (HSD). Significance was expressed as OR and 95% CI. Statistical significance was set at *P* < 0.05. Univariable regression analyses were performed to compare the groups. Subsequently, multiple variable regression analyses were conducted within the same groups to determine whether any individual variable independently achieved statistical significance. Only statistically significant variables (*P* < 0.05) in the univariable regression analyses were included in the multiple variable regression analyses. Analyses were performed using Statistical Package for the Social Sciences (SPSS) v22.

## Results

Fifty patients were enrolled in the study. The median (IQR) age of the cohort was 71 years (57–77), and 30 (60%) were male. The median (IQR) SOFA score on ICU admission was 8 (5–10), and the mean (±SD) SAPS 3 score was 63 ± 11. Eight patients (16%) died within 30 days of admission (Table 1).

**Table 1.** Distribution of baseline characteristic between the MIC-multiple groups on admission to the ICU and during the first 48 h

	Total (*n* = 50)	MIC-multiple of <1 (*n* = 16)	MIC-multiple of 1–8 (*n* = 19)	MIC-multiple of >8 (*n* = 15)	*P* value
Age (years)	71 (57–77)	61 (30–76)*	71 (63–77)	74 (69–81)*	**0**.**050**^b^
Gender, male	30 (60)	12 (75)	11 (58)	7 (47)	0.280^c^
BMI	26 (24–31)	29 (26–32)	28 (24–32)	26 (24–29)	0.393^b^
Obese (BMI > 30 kg/m^2^)	16 (32)	7 (44)	6 (32)	3 (20)	0.389^c^
CCI	4 (2–6)	2.5 (0–4)*	3 (3–7)	4 (4–7)*	**0**.**035**^b^
SOFA on admission	8 (5–10)	9 (4–11)	8 (7–10)	8 (6–9)	0.908^b^
SOFA 24 h, (*n* = 45)	9 (7–10)	8 (6–10)	9 (7–10)	9 (9–10)	0.158^b^
SOFA 48 h, (*n* = 31)	8 (6–10)	8 (4–10)	7 (3–11)	9 (7–10)	0.286^b^
SAPS on admission	63 (±11)	58 (±14)	64 (±8)	66 (±9)	0.203^d^
Septic shock	36 (72)	10 (63)	14 (74)	12 (80)	0.626^e^
Focus of infection					0.172^e^
Pulmonary	20 (40)	9 (56)	7 (37)	4 (27)	
Urinary tract	15 (30)	2 (13)	7 (37)	6 (40)	
Skin or soft tissue	5 (10)	1 (6)	2 (11)	2 (13)	
Abdominal	4 (8)	1 (6)	0 (0)	3 (20)	
Unknown	5 (10)	2 (13)	3 (16)	0 (0)	
CNS	1 (2)	1 (6)	0 (0)	0 (0)	
Creatinine on admission (µmol/L)	135 (111–205)	124 (76–180)	135 (103–290)	180 (128–200)	0.310^b^
Creatinine 24 h (µmol/L), *n* = 31	122 (75–193)	75 (64–119)*	118 (88–196) ¤	184 (154–239)*¤	**<0**.**001**^b^
Creatinine 48 h (µmol/L), *n* = 31	109 (67–207)	65 (57–124)*	88 (70–163) ¤	214 (148–325)*¤	**<0**.**001**^b^
Lactate at admission (mmol/L)	4.4 (2.7–7.1)	5.5 (3.3–6.8)	4.4 (3.3–6.8)	6.6 (2.7–8.8)	0.137^b^
Antibiotic treatment					**0**.**002**^e^
Piperacillin/tazobactam	29 (58)	4 (25)*	12 (63)	13 (87)*	
Cefotaxime	12 (24)	6 (38)	5 (26)	1 (7)	
Meropenem	8 (16)	6 (38)*	2 (11)	0 (0)*	
Benzylpenicillin	1 (2)	0 (0)	0 (0)	1 (7)	
Bolus dose given	22 (44)	5 (31)	9 (47)	8 (53)	0.486^c^
Positive blood culture	21 (42)	6 (38)	8 (42)	7 (47)	0.936^c^
Enterobacterales	11	2	4	5	
*Streptococcus* spp.	7	4	2	1	
*Staphylococcus* spp.	2	0	2	0	
*P. aeruginosa*	1	0	0	1	
Fluid balance Day 1 (*n* = 48)^a^ (L)	3.3 (±2.4)	1.6 (±2.1)*	3.5 (±2.1)	4.6 (±2.4)*	**0**.**003**^d^
Fluid balance Day 2 (*n* = 40)^a^ (L)	0.7 (±1.5)	-0.04 (±1.2)	0.77 (±1.7)	1.2 (±1.4)	0.077^d^
Noradrenaline at 24 h					**0**.**004**^b^
No noradrenaline	8 (16)	6 (38)*	2 (11)	(0)*	
Noradrenaline <0.1 μg/kg/min	12 (24)	6 (38)	4 (21)	2 (13)	
Noradrenaline > 0.1 μg/kg/min	30 (60)	4 (25)	13 (68)	13 (87)	
Noradrenaline at 48 h (*n* = 49)					**0**.**028**^b^
No noradrenaline	27 (55)	12 (80)*	11 (58)	4 (27)*	
Noradrenaline <0.1 μg/kg/min	5 (10)	0 (0)	3 (16)	2 (13)	
Noradrenaline > 0.1 μg/kg/min	17 (35)	3 (20)	5 (26)	9 (60)	
Mechanical ventilation	17 (34)	5 (31)	6 (32)	6 (40)	0.867^c^
CRRT within 48 h	6 (12)	2 (13)	2 (11)	2 (13)	1.000^e^
ICU LOS (days)	3 (2–6)	2 (1–8)	3 (2–4)	3 (2–12)	0.204^b^
Mortality within 30 days	8 (16)	2 (13)	3 (16)	3 (20)	0.894^e^
Mortality at the ICU	5 (10)	1 (6)	2 (11)	2 (13)	0.855^e^

Data are expressed as *n* (%), median (IQR) or mean (±SD). Significant difference in *post hoc* analyses between <1× MIC and >8× MIC groups (*), between <1× MIC and 1–8× MIC groups (#), and between 1–8× MIC and >8× MIC groups (¤). *P* value significant if <0.05.

^a^Patients spent different amount of time in the ICU during these days depending on time of admission.

Statistical methods used to calculate *P* value:

^b^Kruskal–Wallis test.

^c^Pearson’s chi-squared test.

^d^One-way ANOVA.

^e^Fisher–Freeman–Halton exact test.

Positive blood culture results were detected in 21 patients (42%). *Escherichia coli* was the most common pathogen (38%), followed by *S. pneumoniae* (14%). One patient had bacteria isolated from the blood that were resistant to the β-lactam antibiotic (ESBL-producing *E. coli* treated with cefotaxime). The most common site of infection was the pulmonary system (40%) followed by the urinary tract (30%). Septic shock was present in 36 patients (72%), all of whom were treated with noradrenaline upon ICU admission. An additional bolus dose of antibiotics was administered between the first and second doses to 22 patients (44%), 17 of whom had septic shock. β-Lactam antibiotic concentrations were analysed a median of 7 times (range 3–7) per patient (Table 1).

The antibiotic treatment regimen was altered in 10 patients. Specifically, seven patients initially receiving cefotaxime were transitioned to piperacillin/tazobactam (three patients), benzylpenicillin (one patient) and meropenem (three patients). One patient was administered a single dose of piperacillin/tazobactam, which was subsequently replaced with cefotaxime. Additionally, two patients on benzylpenicillin were switched to cefotaxime. For three patients receiving piperacillin/tazobactam, the dosing frequency was increased from every 8 h to every 6 h. Conversely, the dosing frequency was reduced for five patients, with piperacillin/tazobactam adjusted from every 6 h to every 8 h, and meropenem similarly adjusted from every 6 h to every 8 h.

### Antibiotic concentration measurements

Piperacillin/tazobactam was administered to 29 (58%) patients, cefotaxime to 12 (24%), meropenem to 8 (16%) and benzylpenicillin to 1 (2%). An additional single dose of aminoglycosides was administered to 31 patients (62%) on admission.

A total of 294 individual measurements of β-lactam concentration were performed. Of these, 37 measurements were deemed invalid and were therefore excluded (22 with delta time of >8 h after dosing, 13 with uncertain delta time, and 2 with unreasonably high or low concentrations in relation to sampling time, indicating errors in sampling or measurements), leaving 257 samples for analysis (piperacillin, *n* = 153; cefotaxime, *n* = 60; meropenem, *n* = 38; and benzylpenicillin, *n* = 6). The concentration measurements ranges were 2.4–303.4 mg/L for piperacillin, 0.21–29.7 mg/L for cefotaxime, 0.04–31.1 mg/L for meropenem and 83.3–115.5 mg/L for benzylpenicillin.

**Figure 2.**
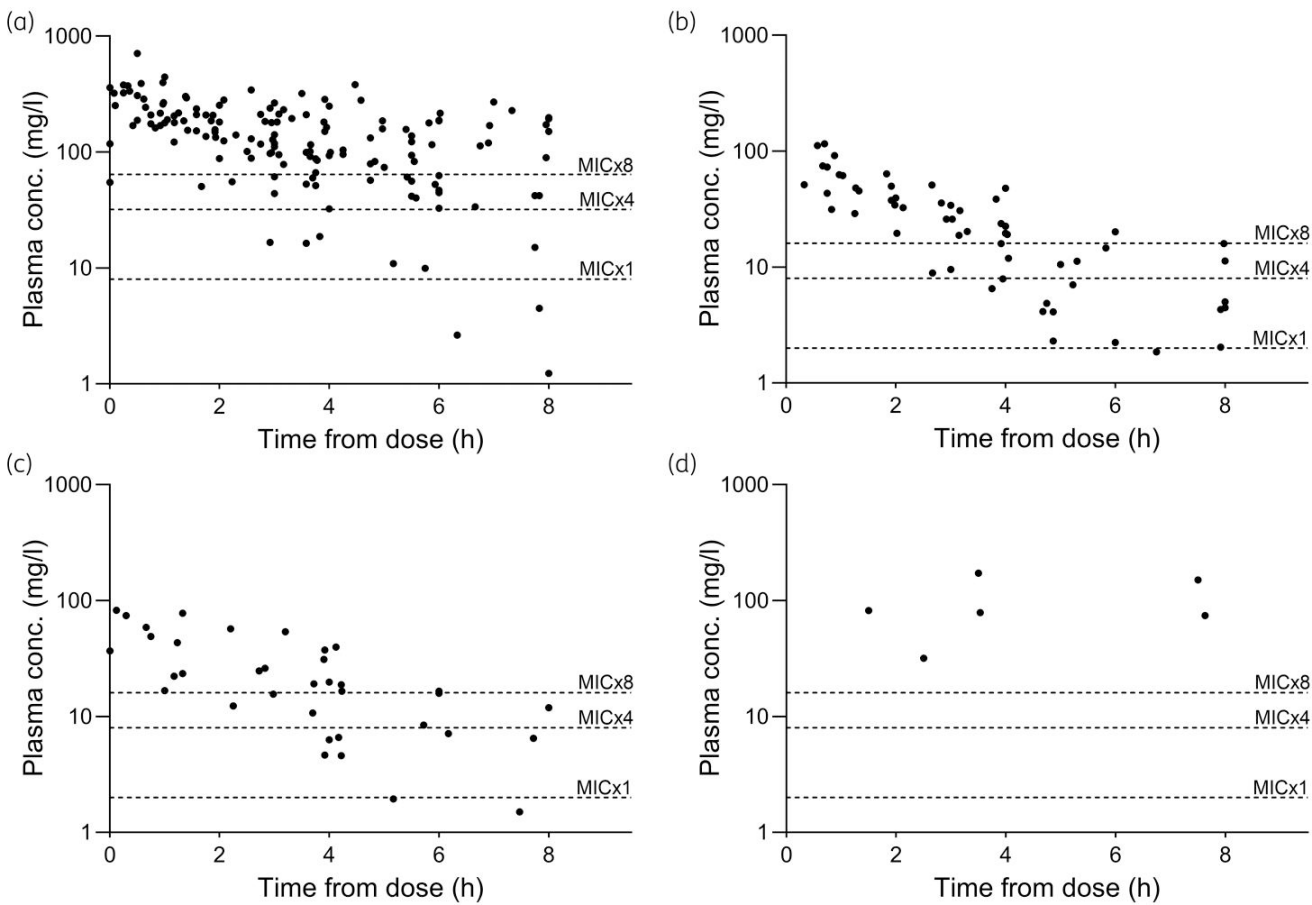
Plasma concentrations of β-lactams related to time from antibiotic dose to blood sampling. (a) Piperacillin (*n* = 153); (b) cefotaxime (*n* = 60); (c) meropenem (*n* = 38); (d) benzylpenicillin (*n* = 8). Target plasma concentrations are shown as dotted lines at ×1 MIC, ×4 MIC and ×8 MIC.

The number of plasma concentration measurements that were <1× MIC at any timepoint during the 48 h period was 15 (3 for piperacillin, 2 for cefotaxime and 10 for meropenem) originating from nine patients. The shortest delta times for these nine measurements were 3.9 h (meropenem), 6.3 h (piperacillin) and 6.8 h (cefotaxime). Owing to the sampling schedule, the results with shorter delta times were more abundant (Figure 2)

The corresponding number when using MIC_ECOFF_ was seven (three for piperacillin and four for meropenem). As these patients were initially empirically treated it is reasonable to consider concentrations covering the highest possible MIC defining the S or I breakpoints for the target pathogen (as clinical breakpoints should predict the clinical effect) and not only the ECOFF (which might be the same MIC but often lower). The factors associated with not reaching target were thus analysed on the MIC_S/I_.

### MIC-multiples and group comparisons based on MIC_s/I_

Sixteen patients displayed an MIC-multiple of <1, 19 patients had an MIC-multiple of 1–8 and 15 patients had an MIC-multiple of >8.

Group comparison showed that younger age, lower CCI at admission, lower serum creatinine concentrations at both 24 and 48 h after admission, lower positive fluid balance on Day 1, lower doses of noradrenaline at both 24 and 48 h after admission, and treatment with meropenem were associated with an MIC-multiple of <1 compared with the other groups (Table 1). Univariable regression analysis comparing patients with an MIC-multiple of <1 with those with an MIC-multiple of >1 confirmed these associations and also found that lower SAPS 3 and treatment with cefotaxime or meropenem were associated with MIC-multiples of <1 (Table 2). In a multiple variable regression analysis, younger age [OR 0.84 (95% CI 0.705–0.991), *P* = 0.039], lower SAPS 3 [OR 1.30 (1.026–1.643), *P* = 0.030] and lower doses of noradrenaline at 24 h [OR 0.01 (0.000–0.671), *P* = 0.034] were independently associated with an MIC-multiple of <1, while lower serum creatinine concentration at 24 h after admission [OR 0.98 (0.957–1.002), *P* = 0.068] was not (Table 2). The independent variable of antibiotic treatment was not included in the multiple regression analysis due to insufficient sample size within each category of this variable.

**Table 2.** Univariable regression analysis of each variable (left) and multiple regression analysis (right) of the group with MIC-multiple of 8

Variables	OR (95% CI)	*P*	OR_adj_ (95% CI)	*P*
Age (years)	0.950 (0.915–0.987)	**0**.**008**	0.836 (0.705–0.991)	**0**.**039**
SAPS	0.941 (0.887–0.998)	**0**.**042**	1.298 (1.026–1.643)	**0**.**030**
Creatinine at 24 h (µmol/L)	0.981 (0.967–0.995)	**0**.**008**	0.979 (0.957–1.002)	0.068
Creatinine at 48 h (µmol/L)	0.983 (0.970–0.997)	**0**.**019**		
CCI	0.748 (0.576–0.973)	**0**.**030**	0.950 (0.542–1.667)	0.858
Fluid balance Day 1 (L)	0.542 (0.351–0.837)	**0**.**006**	0.776 (0.395–1.524)	0.462
Noradrenaline at 24 h	Ref. 0 µg/kg/min			
<0.1 µg/kg/min	0.333 (0.047–2.366)	0.272	0.129 (0.004–3.857)	0.237
≥0.1 µg/kg/min	0.051 (0.008–0.348)	**0**.**002**	0.005 (0.000–0.671)	**0**.**034**
Antibiotic treatment	Ref. piperacillin/tazobactam			
Cefotaxime	6.250 (1.330–29.371)	**0**.**020**		
Meropenem	18.750 (2.757–127.513)	**0**.**003**		

OR_adj_, OR adjusted.

Group comparison revealed that older age, higher CCI, higher serum creatinine concentrations at 24 and 48 h after admission, higher positive fluid balance on Day 1, treatment with piperacillin/tazobactam, and higher noradrenaline doses at both 24 and 48 h were associated with MIC-multiples of >8 compared with the other groups (Table 1). Univariable regression analysis showed that patients with an MIC-multiple of >8 compared with patients with an MIC-multiple of <8 had higher creatinine at both 24 and 48 h, higher positive fluid balance on Day 1, and received higher noradrenaline doses at both 24 and 48 h after admission. The group with MIC-multiples of >8 was also associated with piperacillin/tazobactam treatment (Table 3). Multiple variable regression analysis showed that only serum creatinine level at 24 h [OR 1.02 (95% CI 1.002–1.032), *P* = 0.024] was independently associated with MIC-multiples of >8 (Table 3). As none of the patients treated with meropenem was classified into the MIC-multiple of >8 group, the variable of antibiotic choice was excluded from multiple variable regression analysis.

**Table 3.** Univariable regression analysis of each variable and binomial multiple regression analysis between MIC-multiple of >8 versus MIC-multiple of <1 and MIC-multiple of 1–8

Variables	OR (95% CI)	*P* value	OR_adj_ (95% CI)	*P* value
Age (years)	1.046 (0.996–1.099)	0.071	1.061 (0.987–1.141)	0.110
Creatinine 24 h (µmol/L)	1.015 (1.004–1.026)	**0**.**006**	1.017 (1.002–1.032)	**0**.**024**
Creatinine 48 h (µmol/L)	1.019 (1.007–1.031)	**0**.**002**		
Fluid balance Day 1 (L)	1.432 (1.054–1.945)	**0**.**022**	1.132 (0.755–1.696)	0.548
Noradrenaline 48 h	Ref. 0 µg/kg/min			
<0.1 µg/kg/min	3.833 (0.479–30.7)	0.206	5.754 (0.50–66.256)	0.160
≥0.1 µg/kg/min	6.469 (1.554–26.932)	**0**.**010**	4.695 (0.714–30.867)	0.108
Antibiotic treatment	Ref. piperacillin/tazobactam			
Cefotaxime	0.112 (0.13–0.984)	**0**.**048**		
Meropenem	Not applicable			

OR_adj_, OR adjusted. Only variables with significant results are presented.

## Discussion

This prospective study offers valuable insights into the variability in β-lactam antibiotic concentrations in patients with community-acquired infections during the early stages of sepsis and septic shock. Characteristics associated with an increased risk of low or high antibiotic concentrations were identified. Younger age and lower CCI were associated with failure to maintain 100% of the time above the MIC (<1× MIC). These patients also had significantly lower serum creatinine at 24 and 48 h, lower positive fluid balance on Day 1 and lower SAPS 3, and required less noradrenaline compared with other groups. These findings are consistent with previous research, though few have focused solely on community-acquired infections.^6,7,9,10,23,24^ Interestingly, all patients who received meropenem were in the MIC-multiple of <1 group. When antibiotic choice was excluded, multiple regression analysis revealed that younger age, lower SAPS 3 and reduced noradrenaline levels at 24 h were independently associated with an MIC-multiple of <1. Conversely, the >8× MIC group had higher creatinine, more vasoactive support at 24 and 48 h, and a larger positive fluid balance, indicating a substantial initial impact on multiple organ systems.

Thirty-two percent of the patients exhibited an estimated MIC-multiple of <1 at trough, similar to the percentage reported by Abdulla *et al*. (36%)^7^ but slightly higher than that reported by Smekal *et al*. (23%)^9^ and De Waele *et al*. (19%),^6^ both using the MIC worst-case scenario (MIC_WCS_). Notably, the target attainment rate is heavily influenced by the choice of MIC target.^9^ In this study, we defined the MICs for target attainment to include all isolates classified as S or I, according to the EUCAST breakpoints for species relevant to the choice of specific antibiotics, MIC_S/I_.

This choice was made as only community-acquired infections with sepsis were studied and all empirical knowledge on susceptibility in this group is based on the clinical breakpoints and not the ECOFF. Further, clinical breakpoints are based on a combination of ECOFF, pharmacokinetics/pharmacodynamics (PK/PD) calculations and clinical data supporting clinical efficacy^25,26^ and thus reasonable to aim for as a target..^15,27,28^ As only a minority (42%) of the patients had positive blood cultures, we did not consider MIC_Actual_ as a valid option, but data were analysed using MIC_ECOFF_, resulting in a higher fraction of patients reaching the target of 1× MIC_ECOFF_. The approach of using clinical breakpoints (MIC_S/I_) might thus have overestimated the number of patients with an MIC-multiple of <1 and underestimated those with an MIC-multiple of >8, especially among patients treated with meropenem, where the physician’s decision to use this antibiotic might have been due to suspected meningitis rather than a suspicion of *P. aeruginosa* infection (defining the MIC threshold for meropenem).

All antibiotics were administered as intermittent infusions over 30 min, which could explain a higher number of patients not reaching ≥1× MIC compared with continuous infusion protocols. Previous studies have explored variations in target attainment based on intermittent, prolonged or continuous dosing. Dulhunty *et al*. demonstrated that continuous infusion of β-lactam antibiotics resulted in a higher percentage of time above the MIC^29^ but did not impact 90 day mortality compared with intermittent infusions.^30,31^

At the time of the study, Swedish national guidelines recommended an additional bolus dose between the first and second administration.^32^ Forty-four percent of all patients received a bolus dose, but four still failed to reach >1× MIC. These were all younger male patients with septic shock, low creatinine, short ICU stays and low CCI. These traits have also been associated with risk of underdosing in previous studies.^7,33^ However, as these factors do not explain the risk of underdosing, we believe that future studies should focus on evaluating software to identify other at-risk patients as well.

Despite a wide therapeutic window, elevated β-lactam levels have been linked to neurotoxicity, nephrotoxicity and myelosuppression, although precise toxicity thresholds remain undefined.^23,34^ In this study, high levels were typically seen in older patients with multiple comorbidities and greater organ dysfunction. Confusion, commonly observed in ICU patients with sepsis, may stem from various factors such as fever and hypotension,^12^ but β-lactam toxicity could also contribute.^11,12^ While elevated β-lactam levels in this population are well documented, the underlying mechanisms remain unclear, requiring further research.

Given the substantial pharmacokinetic variability in critically ill patients, individualized dosing strategies are essential. TDM is advised for routine use to tailor antibiotic therapy and detect subtherapeutic concentrations or indicate drug accumulation. In Sweden, widespread implementation of TDM for β-lactams has yet to be established. In hospitals where β-lactam concentration analysis is available, the assay takes approximately 3 h but may not be operational during nights or weekends, limiting clinical utility. Many hospitals rely on transporting samples to external laboratories, resulting in turnaround times that are too long, delaying actionable results.^32^

Although several studies have examined target attainment of β-lactams in critically ill patients, further research is needed to better understand the concentration variability at infection sites.

Most studies, including this one, measured total rather than free drug levels, potentially underestimating target failures. In most cases, the free fractions were estimated from protein-binding assumptions, which may not be accurate in sepsis.^35^

We did not measure or estimate free concentrations in this study. Future work should assess the clinical utility of measuring free versus total levels and improve methods for evaluating unbound drug in sepsis and septic shock.

This study was not powered to assess clinical outcomes relative to MIC-based targets. Larger, ideally randomized, trials are needed to clarify the relationship between exposure, efficacy and toxicity, and to establish PK/PD targets and safety thresholds.

The integration of TDM with model-informed precision dosing (MIPD) offers a promising approach by combining measured drug levels with Bayesian modelling to optimize dosing. This strategy has the potential to improve therapeutic efficacy, reduce toxicity and support antimicrobial stewardship in the ICU. However, further research is needed to assess the optimal model for target attainment and to evaluate the clinical impact and practical implementation of TDM-MIPD approaches in critically ill patients.

A key strength of this study was its prospective design and the inclusion of severely ill patients in the early stages of community-acquired sepsis and septic shock. This allowed detailed assessment of antibiotic concentrations during the initial critical phases. Multiple sampling provided a comprehensive understanding of β-lactam antibiotic PK in each patient for over 48 h.

Owing to the sampling schedule, trough levels were estimated based on three or more actual measurements, in contrast to other studies in which missing values were replaced with values calculated using an appropriate software.^9,36^ The real-world setting in a mixed ICU enhances generalizability.

This study has several limitations. First, since blood sampling was not performed at the trough, the concentration required estimation. We assumed that the antibiotic PK properties followed a one-compartment model, which might have slightly underestimated the trough concentrations. While this may have oversimplified the drug distribution, we still deemed this calculation sufficient, as mainly the last hours of exposure were estimated. Second, the cohort size was limited, and some patients were discharged from the ICU within 48 h, resulting in incomplete data.

### Conclusions

This study found considerable variability in β-lactam concentrations during the first 48 h of community-acquired sepsis. Several patients had concentrations either too low or too high, risking inadequate efficacy and toxicity, respectively. Low antibiotic concentrations were linked to younger age, fewer comorbidities and lower illness severity, whereas unnecessarily high antibiotic concentrations were associated with elevated creatinine and greater organ dysfunction. These findings support the use of TDM in combination with MIPD for early individualized dosing in community-acquired sepsis and septic shock.

## Supplementary Material

dkaf401_Supplementary_Data

## Data Availability

The datasets generated and/or analysed during the current study are available from the corresponding author upon reasonable request.

## Supplementary data

Supplement S1 available as Supplementary data at *JAC* Online.
