# Supplementary material for: β-Lactam concentrations monitored in the early phase of community-acquired sepsis in the intensive care unit
Source: J Antimicrob Chemother. 2025 Oct 28;81(1):dkaf401. doi: 10.1093/jac/dkaf401 (PMC12802928; doi:10.1093/jac/dkaf401)
Supplement: dkaf401_Supplementary_Data [file dkaf401_supplementary_data.docx]

Supplement nr 1.

LCMS/MS

The method employed was fully validated and is used in clinical routine analysis. Calibrators and control samples were prepared by spiking neat substances into blank plasma and were stored as aliquots at -80°C. Four calibrators, and a blank, were prepared from 0.15 to 100 mg/L. Briefly, samples, calibrators, and controls were prepared by pipetting 200 µL of sample into microcentrifuge tubes, adding 20 µL of internal standard, and precipitating with 800 µL of acetonitrile with 0,1% formic acid. The samples were shaken vigorously for 1 min, followed by centrifugation at room temperature for 10 minutes at 13000 g. After dilution of 200 µL of supernatant with 200 µL ultrapure water in glass vials, the samples were analyzed using LC-MS/MS.

The instrumentation consisted of a Waters Acquity UPLC I-Class system with a binary pump and an FTN injector fitted with a sample organizer. A Waters Xevo TQS-µ triple quadrupole mass spectrometer with electrospray ionization was used for detection. Mass Lynx v 4.2 software was used to control the instrument and process the data. The injection volume was 1 µL, and the chromatographic column was an Acquity HSS T3 (1.8 µm 50 × 2.1 mm) column. A gradient of A: 0.1% formic acid in 18.2 MΩ ultrapure water and B: methanol with 0.1% formic acid, starting at 5% B, increasing linearly to 100% B over 2 min followed by a hold and re-equilibration with a total run time of 3 min. Flowrate was 0.6 ml/min. Individual isotope-labeled internal standards were used for all analytes, except for flucloxacillin, where cloxacillin was used. In-process control samples typically show a long time CV<5% both at low (0.3 mg/L) and high (50 mg/L) concentration for all analytes.
